# Mass Spectrometry-Based Metabolomics in Pediatric Health and Disease

**DOI:** 10.3390/metabo16010049

**Published:** 2026-01-06

**Authors:** Debasis Sahu, Andrei M. Matusa, Alicia DiBattista, Bradley L. Urquhart, Douglas D. Fraser

**Affiliations:** 1 Pediatrics, Western University, London, ON N6A 3K7, Canada; debasissahu.phd@gmail.com; 2 Physiology and Pharmacology, Western University, London, ON N6A 3K7, Canada; amatusa@uwo.ca (A.M.M.); brad.urquhart@schulich.uwo.ca (B.L.U.); 3 Children’s Health Research Institute, London, ON N6A 5W9, Canada; 4 Children’s Hospital of Eastern Ontario, Ottawa, ON K1H 8L1, Canada; adibattista@cheo.on.ca; 5 Clinical Neurological Sciences, Western University, London, ON N6A 3K7, Canada; 6 London Health Sciences Centre Research Institute, London, ON N6A 5W9, Canada; 7 Clinical Pharmacology, Western University, London, ON N6A 3K7, Canada

**Keywords:** mass spectrometry, metabolomics, pediatric diagnostics, personalized care

## Abstract

Mass spectrometry-based metabolomics is a valuable tool for advancing pediatric health research. Along with nuclear magnetic resonance, it enables detailed biochemical analysis from minimal sample volumes, a critical feature for pediatric diagnosis. Metabolomics supports early detection of inherited metabolic disorders, monitors metabolic changes during growth, and identifies disease markers for a range of conditions, including metabolic, neurodevelopmental, oncological, and infectious diseases. Integrating metabolomic data with genomic, proteomic (i.e., multi-omics approaches), and clinical information enables more precise and preventive care by enhancing risk assessment and informing targeted treatments. However, routine clinical use faces several challenges, including establishing age- and sex-specific reference ranges, standardizing sample collection and processing, ensuring consistency across platforms and laboratories, expanding reference databases, and improving data comparability. Ethical and regulatory issues, including informed consent, data privacy, and equitable access, also require careful consideration. Advances in high-resolution and single-cell metabolomics, artificial intelligence for data analysis, and cost-effective testing are expected to address these barriers and support broader clinical adoption. As standards and data-sharing initiatives grow, metabolomics will play an increasingly important role in pediatric diagnostics and personalized care, enabling earlier disease detection, improved treatment monitoring, and better long-term outcomes for children.

## 1. Introduction

Mass spectrometry (MS)-based metabolomics is a powerful technology that enables the characterization of the metabolome, the comprehensive set of small molecules, including amino acids, lipids, organic acids, and sugars, found in a biological system [1]. Metabolites are the end products of upstream genetic, transcriptomic, and proteomic processes. They offer a real-time snapshot of an organism’s physiological state and reflect its environment, diet, and disease [2,3]. MS-based metabolomics offers high sensitivity and specificity, enabling analysis of complex biological samples from small volumes collected via minimally invasive methods (e.g., capillary blood) or non-invasive methods (e.g., urine, exhaled breath). This approach is particularly valuable in pediatric research and clinical settings [4].

### 1.1. Metabolomics Is Uniquely Valuable in Pediatrics

Metabolomics is an advanced analytical technique that profiles low-molecular-weight molecules (<1500 Da), such as sugars, lipids, small peptides, vitamins, and amino acids, in biological samples to characterize phenotypes. Its key advantages include high diagnostic accuracy, rapid detection of metabolic changes, and increasingly user-friendly operation. Metabolic fingerprints typically include multiple metabolites and are generated using methods such as nuclear magnetic resonance spectroscopy (^1^HNMR) and mass spectrometry coupled to either gas chromatography (GC-MS) or liquid chromatography (LC-MS) [5]. A unique application of metabolomic technologies in the pediatric population is the screening for inborn errors of metabolism (IEMs). Inborn errors of metabolism are disorders resulting from perturbations in metabolic pathways due to specific genetic mutations that lead to deficiencies in the activity of single enzymes. These conditions contribute significantly to neonatal and infant mortality worldwide. To address this, many countries have implemented newborn screening (NBS) programs, which screen for common IEMs by collecting and analyzing dried blood spots between 24 and 72 h after birth [6].

### 1.2. Pediatric-Specific Considerations

#### 1.2.1. Developmental Metabolic Changes from Birth to Adolescence

Puberty involves hormonal and metabolic changes, such as reduced insulin sensitivity and altered cardiometabolic risk factors. Metabolomic profiling of children across developmental stages can verify healthy metabolic progression or detect early signs of developmental issues, facilitating earlier diagnosis and preventive care. For example, in children with obesity, puberty may trigger a shift from a metabolically healthy to an unhealthy state, reflecting differences in glucose, amino acid, and lipid metabolism [7,8]. These early indicators of diabetes-related cardiometabolic complications often appear during this period and can be detected using high-throughput analytical techniques, such as LC-MS [7,9,10].

#### 1.2.2. Ethical Considerations in Pediatric Research

Although ethics remains central to all research, additional factors must be considered when investigating pediatric populations. The Code of Federal Regulations (CFR) Subpart D establishes ethical standards and protections for child participants in research, acknowledging their specific vulnerabilities. It requires that research involving children generally poses no more than minimal risk and typically includes surveys, observational studies, or retrospective data reviews. The Belmont Report’s principle of respect for persons extends protection to individuals with diminished autonomy, including children, prisoners, and unborn children. Research that is not otherwise approvable may proceed if it addresses a significant issue affecting children’s health or welfare. Parental or guardian permission and child assent are required [11,12,13].

#### 1.2.3. Sample Collection Challenges and Limitations

Collecting samples from pediatric patients presents unique challenges due to their smaller (physical) size, limited blood volume, and the need for specialized techniques. Proper training and coordination between healthcare providers and laboratory staff are essential. Small and fragile veins in pediatric patients often require multiple attempts or alternative sampling methods to obtain a successful biospecimen. Additionally, anxiety and resistance in children may necessitate strategies to reduce distress and improve cooperation. Extra care is necessary when handling, transporting, and storing pediatric samples to maintain their integrity, particularly for tests sensitive to degradation [14]. For example, dried blood spots stored at ambient temperatures after collection showed poor medium- and long-term stability and could not provide reproducible metabolic profiles [15]. Specifically, the stability of acylcarnitines in neonatal dried blood samples was significantly reduced when stored at room temperature [16]. Acylcarnitines were relatively stable when stored at 5 °C for 4 years; however, for optimal stability, samples should be kept at −80 °C for long-term storage. Additionally, minimizing storage time is ideal, as metabolite degradation correlates with storage time, even at −80 °C [17]. Thus, the handling, transportation, and storage of pediatric samples remain crucial to maintaining sample quality.

#### 1.2.4. Age-Specific Reference Ranges and Normalization Strategies

Pediatric reference values are essential, as children’s laboratory results change with growth and development. Additionally, exposure to environmental conditions (i.e., exposome) can also influence pediatric development and the resulting detectable metabolome [18]. Currently, limited age-specific reference ranges make interpreting pediatric test results challenging. These reference ranges should also address the aforementioned difficulties in collecting samples from children. Establishing accurate, age-specific ranges is critical for effective pediatric care across developmental stages [14].

### 1.3. Scope and Aims of This Review

This review provides a concise overview of MS-based metabolomics in pediatric health and disease, highlighting current applications, methodologies, and future directions.

### 1.4. Perinatal and Neonatal Considerations

Metabolomics enables comprehensive analysis of metabolites produced by the body and its microbial communities. This approach is promising for perinatal research, including conditions such as hypoxic–ischemic encephalopathy, intrauterine growth restriction, congenital infections, genetic and metabolic diseases, and neonatal nutrition. In particular, advancing our understanding of the biological and molecular changes in perinatal asphyxia and hypoxic–ischemic encephalopathy serves to improve diagnostic and therapeutic tools. In these cases, positive outcomes are associated with timely detection and treatment; thus, a better molecular understanding results in improved tool sensitivity, specificity, and the ability to detect subtle signals in a timely manner [19,20]. Metabolomics can help identify specific metabolic profiles associated with a particular disease state, disease mechanisms, treatment responses, and outcomes. It may also help neonatologists better detect nutritional deficiencies in key macro- and micronutrients, providing valuable insights into infant nutrition [21].

## 2. Technical Foundations of MS-Based Metabolomics

### 2.1. MS-Based Platforms and Instrumentation

Mass spectrometry (MS) and nuclear magnetic resonance (NMR) are the primary tools for small-molecule analysis in metabolomics. MS, which detects the mass-to-charge ratio (m/z) of analyte ions, in particular, has become increasingly prominent due to its high sensitivity in detecting, quantifying, and elucidating the structure of hundreds of metabolites in a single run from small sample volumes (i.e., often less than 100 µL). Standard separation techniques paired with MS include high-performance liquid chromatography (HPLC), gas chromatography (GC), and capillary electrophoresis (CE). Combined with new software and databases, these hyphenated approaches now support sensitive, automated and quantitative analysis of hundreds to thousands of metabolites. LC and GC are most widely used, while CE is attracting growing interest in the field [3].

#### 2.1.1. Separation Modalities for MS-Based Metabolomics

Liquid Chromatography–Mass Spectrometry (LC-MS) is a versatile technique for detecting a wide range of metabolites with or without chemical derivatization. LC separates compounds based on polarity (e.g., normal-phase, reverse-phase, or HILIC), size (e.g., size-exclusion columns), or charge (i.e., ion-exchange) using a liquid mobile phase; the compounds are then ionized by the mass spectrometer and detected [22,23]. Gas Chromatography–Mass Spectrometry (GC-MS) is often the preferred method for analyzing less polar and volatile metabolites. Analytes are derivatized, injected into the sample port and vaporized. GC-MS employs gas as the mobile phase, requires compound derivatization, and uses standard libraries such as NIST23 (representing the NIST Mass Spectral Library, developed by the National Institute of Standards and Technology (NIST), Gaithersburg, MD, USA) to identify compounds by their mass spectra and retention indices [24]. Capillary Electrophoresis-Mass Spectrometry (CE-MS), specifically capillary zone electrophoresis (CZE), is an effective method for analyzing polar, hydrophilic, and ionogenic metabolites in volume-restricted samples [25]. Advancements have improved sensitivity, which is crucial for pediatric studies with small sample volumes, but issues such as high-pH corona discharge remain.

#### 2.1.2. HRMS (Orbitrap, Q-TOF, FTICR) vs. Triple-Quadrupole/Tandem MS

High-resolution mass spectrometry (HRMS) instrumentation (e.g., Orbitrap, Q-TOF, FTICR) provides high resolution and high mass accuracy for scanned samples. HRMS is ideal for untargeted, discovery-based, complex mixture analysis, structural elucidation, and high-throughput studies. Modern HRMS instrumentation approach triple quadrupole (QqQ) mass spectrometers with respect to quantitative performance (e.g., linear dynamic range), ensuring high-quality, qualitative and quantitative work. However, compared to triple quadrupole mass spectrometers, HRMS approaches have a slower scan rate, seemingly lower sensitivity, but a higher mass resolution. Triple quadrupole (QqQ) mass spectrometry, an instrument built for tandem mass spectrometry (MS/MS), is a platform that offers highly selective and more cost-effective targeted quantification. Compared with triple-quadrupole (QqQ) MS operated in SRM/MRM (selected- or multiple-reaction monitoring), HRMS platforms (e.g., Orbitrap/Q-TOF/FTICR) primarily differ by offering high mass resolving power and accurate-mass selectivity [26]. HRMS sensitivity is therefore not intrinsically lower; in fact, it can be comparable or even superior in some applications (e.g., GC–Q-Orbitrap full-scan reported lower LODs for 86% of analytes than GC–QqQ MRM). The main distinction is that QqQ MS has higher sensitivity for targeted quantification due to ion focusing, despite HRMS having a higher mass resolution. HRMS can also provide stronger confirmatory capability by enabling a higher number of marker ions/peptides/transitions with fit-for-purpose LOD/LOQ (e.g., QTOF identified more marker peptides/transitions for casein and ovalbumin in wines than QqQ) [27]. It is widely used for routine quantitative analysis in complex matrices and has very high sensitivity and a wide dynamic range. While HRMS delivers high-resolution, accurate-mass measurements and has been traditionally used for targeted and untargeted analyses, QqQ is optimized for targeted quantitation. Advances in both HRMS and QqQ have made both suitable for sensitive, reliable quantitation, depending on the application [28].

#### 2.1.3. Mass Spectrometry for Other Omics Platforms

Beyond metabolomics, mass spectrometry is widely applied across other omics platforms, including proteomics, lipidomics, and glycomics [29,30,31]. In proteomics, MS workflows are commonly organized around the biological question being addressed: quantitative proteomics to measure global protein abundance across conditions, post-translational modification (PTM) proteomics to map and quantify modified peptides (e.g., phosphorylation, glycosylation, acetylation), and protein–protein interaction (PPI) proteomics/interactomics to characterize complexes and interaction networks (often including structural MS strategies such as crosslinking-MS or related approaches) [30]. Collectively, these MS-based proteomics strategies complement metabolomics by providing protein-level context for pathway activity and disease mechanisms.

### 2.2. Acquisition Strategies: Targeted vs. Untargeted Workflows

Metabolomics studies can be classified as either targeted or untargeted. Targeted metabolomics, also known as metabolic profiling, detects and quantifies predefined compounds, often those involved in a biochemical pathway. Results can then be age- and sex-matched and compared against reference ranges. Targeted metabolomics is used for screening and diagnosing inborn errors of metabolism as part of newborn screening programs. These methods target known metabolites of perturbed metabolic pathways, including organic acids, amino acids, and acylcarnitines. Targeted approaches are hypothesis-driven, often involve multiple, sequential assays, and are commonly conducted using QqQ mass spectrometry. Accordingly, targeted metabolomics are usually not suitable for compound discovery. In contrast, untargeted metabolomics seeks to detect and identify as many metabolites as possible in a single sample, producing a comprehensive metabolomic profile for a given biochemical phenotype. Untargeted approaches are therefore hypothesis-generating and often employ multiple chromatographic methods and advanced bioinformatics pipelines, including multivariate statistical analyses, to identify biologically relevant compounds unambiguously. As newborn screening programs look to incorporate more omics technologies, untargeted metabolomics shows great promise for characterizing variants of uncertain significance (VUS) identified through whole exome or genome sequencing [32].

Key challenges in untargeted MS-based metabolomics relate primarily to compound annotation and confident identification. Database-driven annotation depends strongly on the scope and curation of reference resources. For example, the Human Metabolome Database version 5.0 (HMDB 5.0, University of Alberta (Wishart Research Group), Edmonton, AB, Canada) aggregates human metabolite records that include chemical structures/identifiers, biochemical context (pathways, reactions), tissue/biofluid locations and concentrations, disease associations, and reference spectra (including MS/MS, GC–MS, and NMR where available) [33]. However, MS/MS spectra alone are frequently insufficient to uniquely distinguish structural isomers and stereoisomers, making orthogonal evidence (e.g., chromatographic separation/retention time, ion mobility-derived collision cross section (CCS), NMR, or comparison to authentic reference standards) necessary for high-confidence identification [34]. In addition, metabolomics lacks a universally adopted, routine analog of proteomics-style identification scoring, and reliable false discovery rate (FDR) estimation remains non-trivial due to chemical diversity and challenges in decoy generation [35,36]. Finally, the quality of fragmentation and library matchability depend on acquisition parameters such as collision/fragmentation energies and instrument-specific settings. This highlights the importance of reporting acquisition details and validating major findings with suitable standards, following community guidelines for assessing identification confidence, such as MSI-style tiered reporting.

### 2.3. Complementary Technologies: NMR, Isotope Tracing, Imaging MS

Nuclear Magnetic Resonance (NMR) spectroscopy is a key complementary analytical tool in metabolomics. While NMR is less sensitive than mass spectrometry, it is non-selective, offers high reproducibility, high accuracy, and can identify unknown metabolites in complex mixtures. NMR, like MS approaches, can trace metabolic pathways and measure metabolic fluxes using stable isotope-labeled precursors. Importantly, NMR metabolomics is non-destructive when compared to MS approaches (which inject samples into the column and ionize them into the mass spectrometer); thus, samples in NMR metabolomics can be reused. This is particularly useful in time-course studies, where samples measured at different time points can monitor metabolic changes over time [37]. Additionally, because NMR does not chemically alter or consume its samples, subsequent NMR analyses can be performed under different conditions (pulse sequences, temperatures) or even using other techniques, such as MS.

Overall, NMR detects metabolites through the magnetic dipole moment of atomic nuclei, including 1H, 13C, 31P, and 15N. Micro-coil probes enhance detection sensitivity by dramatically improving a sample’s signal-to-noise ratio, making NMR suitable for samples with low mass. Advances in data acquisition, such as nonlinear sampling and sophisticated data processing, such as forward maximum entropy reconstruction, have significantly reduced the time required to collect complex, high-resolution data. This is particularly beneficial for two-dimensional (2D) experiments, such as Heteronuclear Single Quantum Coherence (HSQC), which correlate 1H and 13C signals to resolve complex mixtures. Furthermore, specialized methods, such as the SOFAST technique, enable rapid 2D data collection in seconds [38,39,40]. All these tools, with their characteristic features of small-volume sample requirements and non-invasiveness, complement MS-based metabolomics research.

### 2.4. Overview of Metabolomics Study Design

**Figure 1:**
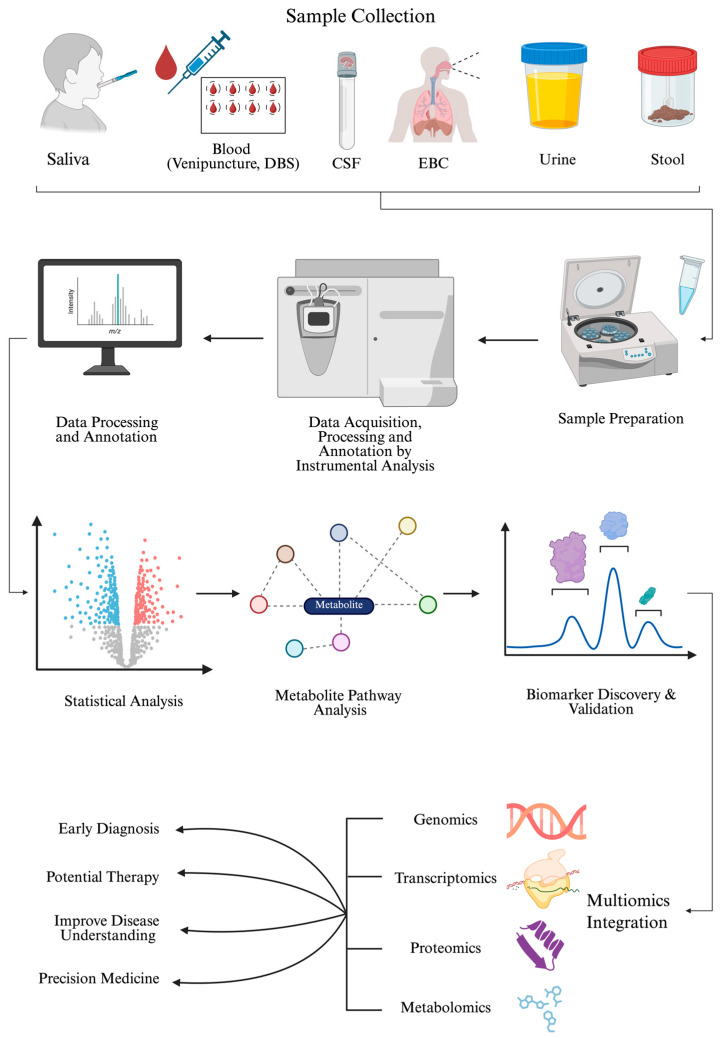
A schematic workflow of a metabolomics study and its clinical impact. This figure illustrates the various steps of a metabolomics study, including the types of samples considered in pediatric cases, their preparation, and instrumental analysis. The data is then preprocessed for statistical analysis to identify significant metabolites. The association of these metabolites with biological pathways is examined, and their relative abundance in diseased patients compared with controls suggests they may serve as potential biomarkers. Newly identified biomarkers are validated in targeted metabolomics assays and used for diagnosis, treatment, disease understanding, and precision medicine, while integrating multi-omics approaches. Abbreviations: DBS (dried blood spot); CSF (cerebrospinal fluid); EBC (exhaled breath condensate). Created in BioRender. Sahu, D. (2026) https://BioRender.com/n4wm8so (accessed on 22 December 2025).

A typical metabolomics study design involves several key steps, beginning with sample collection, storage, preparation, and continuing through sample analysis, statistical analysis, and metabolite identification. Figure 1 outlines the types of samples used in pediatric metabolomics, the sample preparation, and MS-based data acquisition. Once metabolites are detected, the data are preprocessed, MS peaks are annotated, and compounds undergo statistical analysis to identify significant metabolites. All these can be performed using the MetaboAnalyst 6.0 software (Xia Lab, McGill University, Ste. Anne de Bellevue, QC, Canada), which is available in R (version 4.5.2) and as a web application [41]. Subsequent identification and pathway/network analyses can reveal potential biomarkers for use in downstream targeted metabolomics analyses. Finally, integrating additional omics platforms provides a multi-layered understanding of disease phenotypes and subphenotypes, supports early diagnosis, and advances the development of new therapies and precision medicine. In targeted metabolomics studies, matrix-matched standard curves would be generated for each analyte of interest, and an appropriate internal standard would be included in the sample preparation steps. The accuracy and reproducibility of the method are verified by including quality control (QC) samples, which usually reflect the low, middle, and high ends of the reference range for the analytes of interest. In untargeted metabolomics studies where the identity of the metabolite is unknown a priori, a pooled sample is created as a control and injected multiple times throughout the analytical run. The pooled sample consists of a small aliquot of each sample combined and ensures minimal instrument drift across the analytical run.

## 3. Biological Matrices and Pre-Analytical Considerations

### 3.1. Common Pediatric Matrices: Plasma, Serum, Blood/DBS, Urine, Saliva, CSF, Stool, Tears, Exhaled Breath, Sweat

The proper selection, collection, and handling of biological samples are crucial for ensuring high-quality metabolomic data, regardless of the analytical platform or approach used. Table 1 summarizes the various sample types considered in a metabolomics study of pediatric patients, along with their volume ranges, clinical utility, and advantages.

Most research focuses on readily accessible biofluids, such as blood, saliva, cerebrospinal fluid, urine, and feces, with blood as the primary sample in clinical metabolomics [42]. Other samples include exhaled breath, bronchoalveolar lavage fluid, dried blood spots, dried urine spots, or umbilical cord samples [43,44]. Blood-based biofluids, such as plasma, serum, or dried blood spots, are commonly used, as blood is the only biofluid in contact with every organ. Blood-based biofluids capture both blood-native metabolites and transient, inter-tissue metabolites transported in the blood and, as a result, are considered a more comprehensive option, harboring body-wide biomarkers.

The metabolome and lipidome comprise thousands of metabolites that serve as substrates, intermediates, or byproducts of cellular pathways. Additionally, metabolites participate in signaling cascades and the epigenetic regulation of gene expression [45,46]. Therefore, perturbations in these pathways and processes can be associated with disorders in newborns and infants. Targeted metabolomics has formed the core of newborn screening for inborn errors of metabolism since the implementation of triple quadrupole-based mass spectrometry expanded to newborn screening in the early to mid-1990s [47,48,49]. High-resolution mass spectrometry facilitates newer approaches such as biomarker discovery, thereby enhancing predictive and diagnostic capabilities [50]. Precision medicine leverages advances in machine learning algorithms to classify individuals by disease risk or treatment response, supporting a shift toward prevention-focused healthcare. Metabolomics plays a central role in personalizing diagnosis and treatment by exploring the impact of gene variants and gene-environment interactions. Rigorous guidelines have improved study design (e.g., robust quality controls), yielding high-quality data needed to advance precision medicine and disease prevention. However, extensive prospective studies are required to validate and identify metabolomics-based biomarkers with strong clinical potential [51]. The generation of more metabolomic data refines age-stratified metabolite reference ranges. It improves the accuracy and predictive power of machine learning models that rely heavily on large datasets to uncover complex patterns. Efforts such as the HELIX study established pan-European reference metabolomes for healthy children and demonstrated the complementary value of serum and urine in metabolic profiling [52]. It also revealed demographic and dietary influences, identified a novel association with BMI, and provided a valuable resource for future child health research [18,53,54]. Additional studies have highlighted associations between metabolite levels and factors such as age, sex, BMI, and diet, offering important references for future research [53,55,56,57].

Urinary metabolomics provides a non-invasive approach to identify metabolic pathways associated with age, sex, and disease in children. It supports biomarker identification for conditions such as ADHD, diabetes, and asthma, and provides insights into pediatric neuropsychiatric and developmental research by capturing rapid metabolic changes [54,58,59]. Urinary metabolite profiles vary by age and sex, with distinct signatures in children and adults; specific metabolites also show sex-specific variation in pediatric populations. Although comprehensive pediatric urine metabolomics studies are limited, they can provide important insights into health and development [58]. Therefore, the biofluid selected for a particular study should align with the research objectives and the specific disease under study. Finally, integrating metabolomics datasets from multiple biofluids can expand metabolome coverage and further improve diagnosis and treatment for pediatric diseases [60,61].

While plasma profiles provide systemic information, measuring fecal metabolites, which more closely reflect gut bacterial composition, can better reveal associations related to microbial differences. Future research should integrate fecal and serum metabolite data with microbiome sequencing and neurobehavioral assessments to further investigate the gut microbiome-brain relationship. Additionally, fecal metabolite concentrations may also provide a more accurate measure of gut bacterial function than dietary differences [62,63].

**Table 1:** Sample types and recommended safe volume ranges in pediatrics. This table summarizes different types of invasive and non-invasive interventions, along with their recommended safe pediatric volume ranges, collection methods, and clinical utilities.

Sample Type	Safe Pediatric Volume	Collection Methods	Clinical Utility	Advantages	Limitations	References
Blood	Older children and adults (5 mL), infants (1 mL)	Venipuncture	IEMs, cancer studies	High reproducibility and excellent quantitation	Prone to rapid ex vivo degradation before processing	[64,65,66]
DBS	30 µL–100 µL per spot	Heel prick for neonates, fingerstick for older children	Newborn screening, disease monitoring	It is easy to perform, requires no sample freezing or temperature maintenance before analysis, and is minimally invasive.	Hematocrit-dependent variability affects quantitative accuracy.	[66,67,68]
DUS	15 mL	Whatman 903TM filter paper card	Test for urine creatinine, HPV, study metabolites in HCC, and metabolic syndrome	Improved sample stability	Small spot volume and dried-matrix format increase analytical complexity.	[69,70]
Urine	1–2 mL	Cotton ball or diaper urine pad for infants	Jaundice syndrome, celiac disease (CD), COPD, HCC	Non-invasive	Requires normalization to creatinine to account for dilution.	[18,65]
CSF	100 µL	Lumbar puncture	Neurological diseases	Reflect the brain’s biochemistry	Lumbar puncture is relatively invasive and may be unsuitable for pediatric patients.	[65,71,72,73]
EBC	2–3 mL	Ecoscreen device	Study shock and respiratory failure	Non-invasive	Low metabolite concentrations require highly sensitive methods	[74,75]
Saliva	20 µL	Passive drool collection or swab	Oral cancer, oral precancerous lesions, periodontal diseases, and dental caries	Minimally invasive	Food/drink intake, oral hygiene, medications and other exposures increase variability.	[65,76,77]
Stool	1 g	Stool collection tubes	Microbiota evaluation	Ease of collection	High sample heterogeneity and microbiota influence metabolomic profiles	[78,79]
Umbilical cord	50–100 µL	Pall Medical cord blood collection kit	LBW babies and their mothers	Easy to collect, risk-free for the mother and newborn	Low tissue quantity compared to other sample types	[44,80]

Abbreviations: DBS (dried blood spot); DUS (dried urine strip); CSF (cerebrospinal fluid); EBC (exhaled breath condensate); IEMs (inborn errors of metabolism); LBW (low birth weight); HPV (human papillomavirus); HCC (hepatocellular carcinoma); COPD (chronic obstructive pulmonary disease).

### 3.2. Age-Appropriate Blood Sampling and Safe Volumes

Collected blood volume must align with each patient’s age and weight, in accordance with the established guidelines for each center in which the sample is collected. Particular care must be taken when drawing blood from children and vulnerable patients to ensure they remain within safe limits. Typically, blood draws are restricted to 1% of the total blood volume per day and 3% over four weeks. For pediatric patients in clinical studies, especially those with lower body weight, it is essential to monitor and manage daily blood collection to avoid potential adverse health impacts or overdrawing [81,82].

### 3.3. Collection Timing: Fasting, Circadian, and Feeding Effects

The timing of sample collection, as well as whether the patient is in a fasted or fed state, must be considered, as these factors can significantly affect test results [18]. Therefore, samples should be collected at the same time each day and in a similar state of fasting to reduce variability in the metabolome. Regardless of the study requirements, documentation of collection time, fasting status, and recent eating history is crucial to support accurate results and quality patient care [70,83].

### 3.4. Storage, Transport, and Biobanking Stability

Metabolites must be protected from degradation by enzymatic and non-enzymatic processes [84]. For example, plasma levels of the amino acid homocysteine are known to increase if blood samples are not centrifuged immediately. All samples should be frozen at −80 °C as soon as possible after collection (or processing, if whole blood is spun to plasma or serum), and the cold chain must be maintained during shipping and storage. This is costly and may be impractical for some laboratories or facilities. Ambient-temperature storage is more practical, though most sample types will exhibit significant changes in metabolomic profiles at ambient temperatures. Similarly, freeze–thaw cycles can affect the stability of some metabolites, so careful planning and sample aliquoting are required to minimize their effects [85]. Metabolite stability in dried blood spots (DBS), a sample type typically considered to be stable under ambient conditions, depends on time and temperature: short-term stability is generally good, but medium- to long-term stability varies with storage conditions. At room temperature, most metabolites remain stable in DBS during the first month and stable for at least a year when stored at −20 °C, although some, such as phospholipids and acylcarnitines, degrade by over 30% [86,87]. One study found that over 71% of the metabolome remained stable after 10 years at −20 °C, with lipid-related metabolites declining [88]. These findings support the use of DBS samples for metabolomic studies, though long-term storage at −80 °C and monitoring of specific unstable metabolites is recommended [88,89,90].

### 3.5. Standard Operating Procedures (SOPs) and International Organization for Standardization (ISO) Recommendations

Serum and plasma samples should be frozen immediately after centrifugation at 4 °C. Cold storage at approximately 4 °C is acceptable for up to two hours to preserve sample quality and minimize enzymatic and non-enzymatic changes in the metabolome. For extended storage, a validation study should be conducted to assess the stability of the metabolites of interest under refrigerated conditions. As outlined in ISO 23118, blood-based samples should be thawed on ice before measurement [91]. Further sample processing (i.e., metabolite extraction, dilution, internal standard addition) must begin promptly after the sample has been thawed. Any unused sample should be refrigerated after aliquoting and refrozen immediately. Freeze–thaw cycles should be avoided if possible, but limited to 2 unless additional quality assurance measures confirm reliable metabolite measurements with additional cycles [92].

### 3.6. Practical/Ethical Aspects: Assent/Consent

Children’s consent for medical care should be based on their developmental stage, not simply modeled after adult informed consent. Consent involves helping the child understand their condition, explaining upcoming tests and treatments, assessing their comprehension, any external influences, and confirming their willingness to proceed. Research with children requires written parental or guardian permission, unless the research ethics board/institutional review board (REB/IRB) grants a waiver based on specific criteria focused on protecting the children. A waiver may be permitted when obtaining parental/guardian permission is not reasonable, provided alternative mechanisms are in place to protect the children, and the waiver does not violate the law. Assent, defined as a child’s affirmative agreement to participate, must be obtained according to the child’s age, maturity, and psychological state. The REB/IRB decides whether assent is documented in writing or verbally. If parental or guardian consent is not reasonable for a specific population, such as children in care, the REB/IRB may waive this requirement if an alternative protection mechanism is in place and all applicable laws are followed [12,93].

## 4. Sample Preparation and Analytical Quality Control

Metabolomics research in children, particularly newborns, is challenging due to limited sample volumes and rapid metabolic changes during growth [94]. Researchers often rely on microsampling methods, such as dried blood spots (DBS), or by collecting small volumes of whole blood in pediatric micro-volume tubes, providing less than 100 µL of plasma [95,96]. Standardizing pre-analytical and analytical procedures is essential for obtaining reliable data and subsequent results [97,98]. Technical bias, which can be challenging to detect, compromises reproducibility while introducing and propagating persistent measurement errors [99]. Adhering to standardized protocols for sample collection, handling, processing, and storage helps reduce technical bias and non-biological variability [100]. Selecting a method to extract the metabolites of interest from the sample is non-trivial. The solvents or chemicals used must be compatible with the chosen analytical platform, reproducibly extract metabolites, and minimize the presence of matrix components, such as proteins. Typically, deproteinization and precipitation are performed by adding ice-cold organic solvents such as methanol or a methanol-ethanol mixture, thereby minimizing interference [101,102]. Additionally, robust quality control (QC) strategies are necessary to ensure accurate and consistent measurements, especially in large-scale studies [98]. This includes creating a pooled quality control sample by combining equal amounts from each study sample to provide a broad representation of the entire study population. A QC sample is analyzed sporadically between study samples to monitor and correct instrumental and batch-related drift, thus maintaining data reliability and consistency [103,104]. FAIR principles, NIST reference materials, and MetaboLights (EMBL’s European Bioinformatics Institute (EMBL-EBI), Hinxton, Cambridgeshire, UK) are key international initiatives that aim to standardize and harmonize metabolomics data. The FAIR data principles emphasize that data should be findable, accessible, interoperable, and reusable, offering a framework that enhances transparency and long-term usability of datasets [105]. MetaboLights serves as an open-access repository for metabolomics studies, providing a single point of access to global metabolomics data and knowledge, thereby ensuring data traceability and reproducibility [105]. Additionally, NIST has developed standard reference materials (SRMs), such as SRM 967a for creatinine in frozen human serum, which are vital for achieving inter-laboratory comparability of metabolomics measurements [106].

## 5. Data Acquisition, Processing, and Computational Analysis

Once a sample type has been selected, samples have been collected, shipped, processed, and stored in accordance with best practices, the platform of interest then analyzes the samples. Mass spectrometry is the most commonly used platform in metabolomics, generally coupled to chromatographic separations [107]. Once the data is acquired, the first step is preprocessing, which includes peak picking, spectral deconvolution, chromatographic time alignment, normalization and/or data transformation and scaling. The MS detector output is continuous data (e.g., chromatogram of retention times vs. ion abundances vs. *m*/*z*), which is converted into discrete feature tables that capture signals representing metabolites by accurate mass or *m*/*z*, retention time, fragmentation pattern and whose responses are defined by ion abundances or integrated peak area [108].

Data filtering is typically used to remove chromatographic peaks that are not biological in origin (such as instrumental or random noise). Peaks that are non-reproducible (i.e., coefficient of variation in the QC samples above a predefined value) or derived from the processing steps (e.g., solvents, tips, tubes, filter paper) are also removed at this stage. Statistical analyses can now be performed on the preprocessed and filtered data. Multivariate methods, such as principal component analysis (PCA), are used for data quality control, outlier identification, and to visualize sample clustering. Supervised machine learning techniques, like partial least squares discriminant analysis (PLS-DA), identify metabolites that best distinguish between disease and control groups [109,110]. Advanced machine learning techniques such as Random Forests (RF), Support Vector Machines (SVMs) and Deep Learning (DL) neural networks can be employed to build highly accurate predictive models for complex and subtle molecular patterns; however, it is worth noting that DL methods are computationally exhaustive and, at times unwarranted when simpler machine learning algorithms provide comparable predictive power [111]. Analytes that are significantly different between treatment and control groups should be identified, if possible. Unambiguous identifications are necessary to understand the biochemical significance of these perturbations with respect to phenotypes and can inform downstream, targeted assays. Features are compared with known databases, such as the HMDB 5.0 [112] and Metabolite and Chemical Entity Database (METLIN v1.0.0r00) (Scripps Research, La Jolla, CA, USA) [113]. However, this step highlights a current limitation in metabolomic studies: although databases contain information on thousands of metabolites, several features in an untargeted experiment remain unidentified [114]. Therefore, efforts are required to continue discovering and identifying metabolites to facilitate metabolomics research.

Tools like MetaboAnalyst 6.0, which offers both a web-based graphical user interface and a code-based R package (version 4.0), map metabolite changes to specific biological pathways, such as amino acid or lipid metabolism. Indeed, MetaboAnalyst 6.0 has emerged as a one-stop shop for metabolomics analysis, now able to process MS-spectral data, perform statistical analyses, clustering, pathway- and network-mapping, and multi-omics integration [41]. These analyses, particularly pathway- and network-mapping, help researchers understand how metabolite changes relate to phenotypes and disease [115]. The significant age, sex and intra-individual variation in the metabolome also requires careful consideration. As the pediatric metabolome changes rapidly, advanced models are needed to distinguish disease effects from normal growth and age-related variation [58,116]. Additionally, as laboratories follow different experimental and analytical protocols, it becomes harder to reproduce results, incorporate data from other laboratories in meta-analyses, and use identified biomarkers in clinical practice [117,118]. Therefore, researchers are increasingly applying Artificial Intelligence and longitudinal study designs to address these challenges and accelerate biomarker discovery and validation [119,120,121].

## 6. The Pediatric Metabolome: Development and Reference Resources

Research shows that the pediatric metabolome changes substantially from infancy to adolescence. Studies of healthy children aged 6 months to 4 years found that urinary metabolites vary with age. For example, trimethylamine N-oxide (TMAO) and betaine are higher at 6 months, while glycine and glutamine drop after infancy. Creatine and creatinine increase as children grow, matching their physical and muscle development. Amino acid metabolism is most active in the first year, while carbohydrate metabolism changes more in the second and third years. These results suggest that metabolomic profiles can help track both diet and development. Additionally, maternal health has also been demonstrated to impact the pediatric metabolome, as children born to mothers with obesity or gestational diabetes show lasting changes in serum metabolites, even at ages 10 and 16. Therefore, the pediatric metabolome is dynamic and influenced by age, growth, diet, and early-life factors. This highlights the importance of metabolomics for understanding pediatric development and long-term metabolic health, as well as for understanding perturbations resulting from disease [58,122].

### 6.1. Age-Dependent Metabolic Trajectories (Neonate → Adolescence)

**Figure 2:**
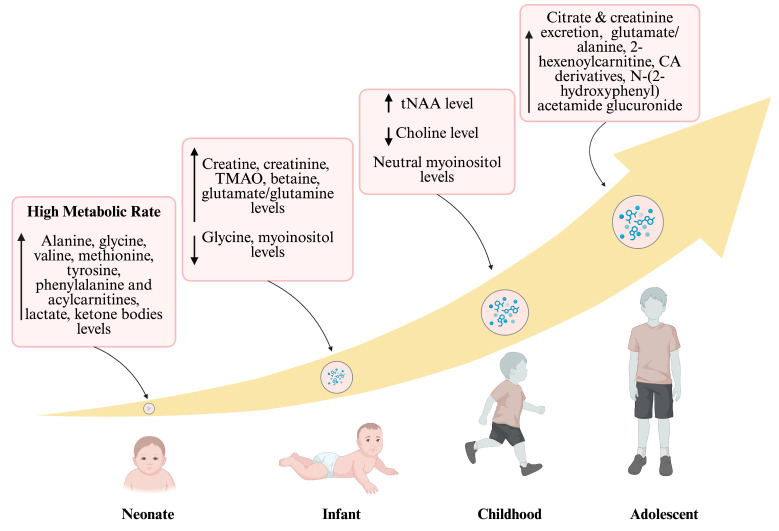
Developmental trajectories of metabolome. This figure summarizes the trajectory of metabolomic shifts from the neonatal period to adolescence. Upward curved arrows represent the increasing age, along with the levels of metabolites displayed in a box. Within each pink box, the upward arrow (**↑**) indicates metabolites that increase and the downward arrow (**↓**) indicates metabolites that decrease in abundance relative to the preceding developmental stage; “neutral” indicates no appreciable change. Neonates show a high metabolic rate and increased levels of amino acids and acylcarnitines. Reduced levels of glycine and myoinositol are observed, while creatine, creatinine, lactate, and glutamate/glutamine levels increase in infants. During childhood, tNAA levels rise, choline levels decrease, and myoinositol remains at a neutral concentration. By adolescence, there is enhanced excretion of citrate, creatinine, and an elevation in carboxylic acid derivatives, as well as TMAO (trimethylamine N-oxide); tNAA (total N-acetylaspartate); and CA (carboxylic acid). Created in BioRender. Sahu, D. (2026) https://BioRender.com/n4wm8so (accessed on 22 December 2025).

Throughout the transition from neonatal stages to adolescence, a significant metabolic shift occurs [123]. Figure 2 illustrates this trajectory, highlighting characteristic metabolites across different age groups. Neonates exhibit elevated levels of lactate and ketone bodies due to brain metabolism, as well as increased levels of amino acids and acylcarnitines. Elevated excretion of creatine and creatinine indicates muscle metabolism waste in infants [124]. During childhood, there are high levels of total N-acetylaspartate (tNAA) combined with decreased choline and neutral myoinositol [125]. By adolescence, there is a notable increase in absolute urine excretion of citrate and creatinine, along with higher levels of hexenoylcarnitine, carboxylic acid derivatives, and N-(2-hydroxyphenyl) acetamide glucuronide. Understanding and monitoring these metabolites are important for early disease detection and monitoring responses [126]. Ultimately, metabolomic trajectories are dynamic displays of the age-dependent changes [58].

### 6.2. Sex Differences and Puberty Effects

Both baseline sex differences and puberty significantly influence the pediatric metabolome. Boys and girls follow distinct metabolic pathways, with sphingolipids accounting for these differences from birth to age 4. Specifically, girls show higher levels of sphingomyelins. The most evident differences in metabolomic profiles occur around one year of age, coinciding with other physiological changes, such as changes in hormone distribution and increased infection risk. In obese adolescents, boys exhibit higher levels of branched-chain amino acids and related metabolites than girls, which are associated with insulin resistance and lipid profiles in sex-specific ways, such as increased triglycerides in boys compared to girls. Puberty is a critical period when hormonal and metabolic changes, particularly an increased insulin resistance, elevate the risk of type 2 diabetes and obesity. Early onset of puberty further heightens these risks. Both sex and the timing of puberty play key roles in determining metabolic health in children [8,127,128,129].

### 6.3. Maternal and Nutritional Influences

Perinatal nutrition is central to the Developmental Origin of Health and Disease (DOHaD) hypothesis, which states that events during the periconceptional period and pregnancy shape long-term health. Metabolomics offers valuable insights into how perinatal nutrition affects mothers and newborns [130]. Suboptimal maternal nutrition, whether insufficient or excessive, can alter adipose tissue, brain development, energy regulation, and organ structure. These changes may result in long-term adaptations that disrupt energy balance and increase the risk of obesity and metabolic syndrome [131]. Mothers with a BMI of 25–29.9 (overweight) or over 30 (obese) had higher lactose, lower orotic acid, 3-indoxyl sulfate, heneicosanoic acid, and N1-methylguanosine in their milk than mothers of normal weight. Lower orotic acid levels were associated with greater infant weight gain. Mothers with low adherence to the Mediterranean diet had reduced citric acid, N6-succinyladenosine, uric acid, and eicosenoic acid, and increased acylcarnitine C6:0, compared to those with higher adherence. Maternal intake of fruit and fish was the most influential dietary factor for healthy early infant growth. These findings highlight the importance of maternal fruit and fish consumption during lactation for optimal infant weight gain and provide further insight into how maternal factors influence breast milk composition and infant growth [132]. Applying a metabolomics approach can provide an in-depth molecular snapshot of an individual’s phenotype, including both healthy and disease states. For example, metabolomics can assess the impact of different nutritional regimens and provide guidance to maximize health, thereby supporting a preventive approach [133].

### 6.4. Existing Pediatric Reference Datasets and Gaps

Comparing patient metabolomic profiles to reference datasets enables phenotype classification and facilitates interpretation of results. One pool of metabolomic data was previously mentioned, the HELIX study, which aimed to establish pan-European pediatric reference metabolomes. Having comprehensive reference datasets remains crucial in pediatric metabolomics research. Blood metabolite levels are affected by factors including gestational age, birth weight, sex, ethnicity, age at blood collection, nutritional therapy, and season of birth, all of which can influence newborn screening accuracy. Adjusting results based on these variables using post-analytical tools, such as Clinical Laboratory Integrated Reports (CLIR) version 2.27.00 (Mayo Clinic, Rochester, MN, USA), enhances screening performance and reduces false positives. Tools like CLIR 2.27.00 aim to replace traditional cutoffs with continuous thresholds and reference ranges that account for demographic and developmental factors. Machine learning algorithms have also been applied to metabolomic screening data, including newborn screening, to reduce false-positive rates further [134]. The Recommended Uniform Screening Panel database and web-based tool version 1.0 (dbRUSP v1.0), Yale University School of Medicine, New Haven, CT, USA, developed using the R Shiny package 1.7.2 (Posit Software, PBC, Boston, MA, USA), analyzed 41 newborn screening metabolites and six variables, birth weight, sex, age at blood collection, gestational age, parenteral nutrition status, and parent-reported ethnicity, in a cohort of 500,539 screen-negative newborns from the California NBS program. The interactive database allows users to examine the effects of individual and combined variables on metabolite levels [135]. While various metabolomic databases are available, the heterogeneity and variance in the pediatric metabolome require the curation of more diverse reference datasets that account for different demographics, developmental stages, genetic variants, and life experiences. Building on the identification of current datasets and their limitations, the following section highlights the importance of reference ranges when interpreting metabolomic data.

### 6.5. Establishing Reference Intervals

Accurate reference intervals are required for clinical laboratory tests to support the interpretation of results and informed decision-making. Establishing these intervals requires large, healthy reference populations, which is especially challenging in pediatrics. Many laboratories continue to use outdated pediatric reference intervals that may not reflect current populations or methodologies. Pediatric intervals must account for physiological changes in biomarker levels across age groups, particularly during infancy and puberty [136,137]. The CLSI EP28-A3c guidelines recommend separate reference intervals by age and sex to address these differences [138]. For example, a meta-analysis of two large, independent, population-based studies found limited evidence for a link between NMR-based metabolomic profiles and subclinical atherosclerosis in children, after adjusting for age and sex [139]. This reinforces the importance of age- and sex-specific reference ranges when interpreting metabolome variation. Therefore, while metabolomics captures broad physiological differences across development, its clinical interpretability depends on robust reference ranges.

## 7. Clinical Applications of MS-Based Metabolomics

### 7.1. Newborn Screening and Inborn Errors of Metabolism

Metabolomics, particularly using tandem mass spectrometry (MS/MS) and gas chromatography–mass spectrometry (GC-MS), has advanced pediatric public health by enabling the detection of metabolic disorders pre-symptomatically. Since its adoption in the 1990s, MS/MS has become the gold standard for newborn screening worldwide. LC-MS/MS has become the standard for newborn screening, as this approach enables rapid, simultaneous detection of a wide range of metabolic and endocrine disorders from a single blood sample, greatly improving early diagnosis and intervention in neonates and infants. As a result, metabolomics has led to newborn screening ranking among the ten most important achievements in public and pediatric health in the early 21st century [140,141,142,143,144,145,146].

Inborn errors of metabolism (IEMs) are inherited disorders resulting from defects in specific metabolic pathways. IEMs are often present as multi-systemic, which can complicate and delay diagnosis. Tandem mass spectrometry can simultaneously screen for more than 30 inborn errors of metabolism from a single dried blood spot sample. Early detection of IEM is crucial to begin treatment to mitigate the adverse health outcomes of disorders such as amino acidemias, urea cycle disorders, organic acidemias, and fatty acid oxidation. MS/MS, without a chromatographic separation, is used for initial screening, while GC-MS and LC-MS/MS help confirm diagnoses.

Because metabolism is enzyme-driven, many metabolic disorders reflect altered enzyme abundance or activity; correspondingly, metabolomics can be interpreted as a functional readout of enzyme perturbation, often evidenced by substrate accumulation and altered substrate/product ratios [147]. For example, phenylketonuria results from a deficiency of phenylalanine hydroxylase (PAH), leading to phenylalanine accumulation; medium-chain acyl-CoA dehydrogenase (MCAD) deficiency reflects insufficient MCAD activity and yields characteristic disruptions in medium-chain fatty acid oxidation; and maple syrup urine disease is caused by a deficiency of the branched-chain α-ketoacid dehydrogenase complex (BCKDC), producing elevations of branched-chain amino acids and their related metabolites. These examples illustrate how metabolite signatures can be mechanistically anchored to enzyme-level dysfunction, strengthening biological interpretation of reported biomarkers [148,149,150].

Approximately 1000 IEMs have been identified, each caused by genetic variants that disrupt proteins essential for cellular metabolism. These disruptions can reduce energy production or cause the accumulation of harmful substances, leading to disease. While there are over 1000 IEMs, newborn screening programs typically screen for 30–75 conditions. This is often because suitable markers (i.e., small molecules amenable to MS/MS detection) are not known or available for all disorders. As a result, additional specialized metabolic laboratory tests are required for diagnosis. Advances in metabolomics, particularly in biomarker discovery and pathway mapping, have enabled the identification of diagnostic biomarkers and the elucidation of new metabolomic patterns in previously unrecognized or under-screened disorders. To complicate matters, clinical symptoms vary significantly, even among individuals with the same disorder. This concept, ubiquitous across biology, is known as heterogeneity, and it remains a core reason why personalized medicine approaches are necessary for efficacious treatment. Thus, metabolomics is now routinely used for newborn screening, differential diagnosis, monitoring disease progression, and treatment response [151,152,153]. Recent advances in gene editing have suggested promising new avenues for treating patients diagnosed with IEM and highlight the importance of early detection. Specifically, CRISPR-Cas9 base editing was recently performed in an infant to correct an IEM using a personalized lipid-nanoparticle approach [154]. This underscores the importance of personalized healthcare while highlighting the potential of advanced molecular therapies, such as base editing, to correct deleterious genetic variants.

**Figure 3:**
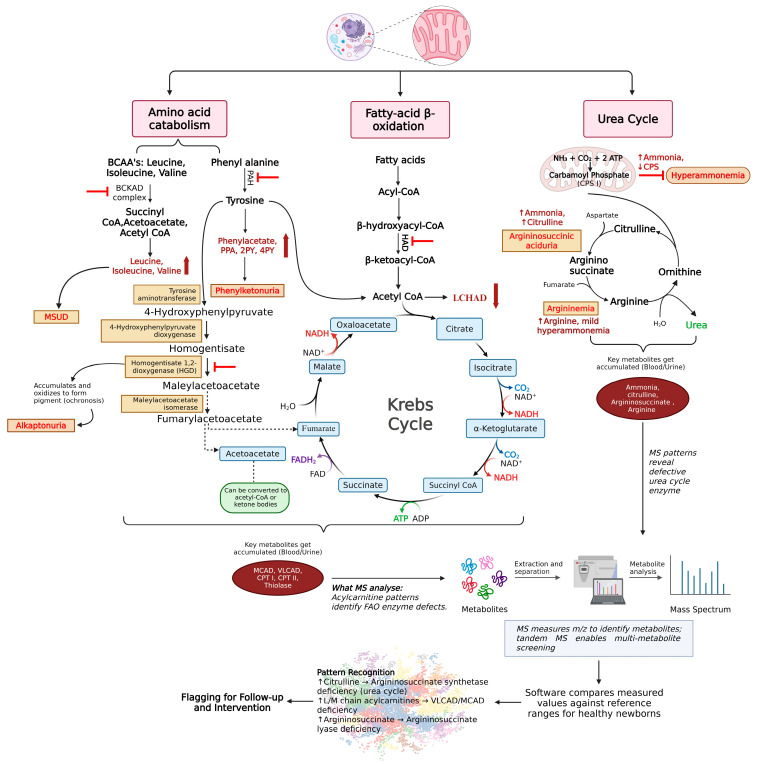
Major metabolic pathways are affected in inborn errors of metabolism. The figure depicts the major metabolic pathways affected in inborn errors of metabolism. It highlights three key mitochondrial pathways that are commonly disrupted in pediatric IEMs: branched-chain amino acid metabolism, fatty acid oxidation, and amino acid and urea cycle pathways. Defects in specific enzymes within these pathways lead to the buildup of characteristic metabolites, indicated by upward-facing red arrows. These elevated metabolites serve as the basis for biochemical diagnosis and disease monitoring. The figure also outlines the general workflow for detecting and identifying metabolites using mass spectrometry (MS), illustrating how pathway-specific metabolic disruptions can be identified through targeted or untargeted metabolomic approaches. Color/symbol key: Upward red arrows indicate increased/accumulated metabolites; red inhibitory bars/block symbols mark the disrupted enzymatic step; gray/black arrows indicate reaction direction and workflow links; blue boxes denote Krebs cycle intermediates; orange boxes denote example IEM diagnoses/phenotypes; pink header boxes label pathway groups. Abbreviations: Branched chain amino acid (BCAA), Branched chain keto acid dehydrogenase complex (BCKAD), Inborn errors of metabolism (IEM), Maple syrup urine disorder (MSUD), Phenylalanine hydroxylase (PAH), Phenyl pyruvic acid (PPA), N-methyl-2-pyridone-5-carboxamide (2PY), N-methyl-4-pyridone-3-carboxamide (4PY), Phenylketonuria (PKU), Long-chain 3-hydroxyacyl-CoA dehydrogenase (LCHAD), and Mass spectrometry (MS), Medium chain acyl CoA dehydrogenase (MCAD), Very long chain acyl CoA dehydrogenase (VLCAD), Carnitine palmitoyl transferase (CPT), Fatty acid oxidation (FAO), Nicotinamide adenine dinucleotide oxidized form (NAD), Nicotinamide adenine dinucleotide reduced form (NADH_2_), Carbamoyl phosphate synthetase (CPS), Mass to charge ratio (*m*/*z*), Flavin adenine dinucleotide (FAD), Reduced Flavin adenine dinucleotide (FADH_2_). Created in BioRender. Sahu, D. (2026) https://BioRender.com/n4wm8so (accessed on 22 December 2025).

Figure 3 highlights key metabolic pathways affected by inborn errors of metabolism, including amino acid catabolism, fatty acid beta-oxidation, and the urea cycle, which all influence the TCA cycle. Enzyme malfunctions in these pathways lead to the accumulation of harmful substances, resulting in metabolic disorders in newborns.

### 7.2. Metabolic and Endocrine Disorders: Obesity, Diabetes and MASLD

Metabolic and endocrine disorders like obesity, diabetes, and metabolic dysfunction-associated steatotic liver disease (MASLD, previously NAFLD: nonalcoholic fatty liver disease) are becoming more common in children and teens, creating serious public health concerns and long-term risks [155]. These conditions often co-occur with metabolic syndrome, high blood pressure, abnormal cholesterol levels, and insulin resistance, making early detection and intervention especially important. In addition to traditional diagnostic tools, new methods such as mass spectrometry-based proteomics and metabolomics now provide detailed molecular profiles. For example, recently identified metabolomic signatures in pediatric patients with diabetic ketoacidosis (DKA) revealed differentially abundant metabolites that correlate with specific clinical features, underscoring the use of omics technologies as a future clinical tool [156,157,158]. Biomarkers, including inflammatory and metabolic markers and gut microbiota profiles, help improve risk assessment, disease monitoring, and personalized treatment. There is also a shift toward using broader health indicators beyond BMI and exploring non-invasive tests, such as salivary metabolomics. Continued research on new biomarkers and gut microbiota is helping improve diagnosis, prognosis, and management, supporting earlier intervention and better outcomes for children with these disorders [77,159,160,161,162].

### 7.3. Pediatric Oncology: Tumor Metabolism, Therapy Monitoring

Metabolomics plays a growing role in pediatric oncology by helping researchers understand how tumors work, facilitating earlier detection, and tracking how children respond to treatments. This approach analyzes the metabolomic profile to show how cancer and its treatments alter metabolism. By identifying unique metabolic patterns and biomarkers, metabolomics enables tumor diagnosis, leading to tailored treatments monitored with non-invasive tools such as positron emission tomography (PET) and magnetic resonance spectroscopic imaging (MRSI) scans. Although it can be difficult to separate disease-related changes from normal differences and to link findings clearly to clinical outcomes, ongoing research is building metabolite atlases and improving methods to make diagnosis, treatment, and prognosis better for children with cancer [5,163,164,165].

### 7.4. Neurology and Neurodevelopment

Metabolomic profiling in children with autism spectrum disorder (ASD) offers potential for improved diagnosis, personalized treatments, and better monitoring of therapy outcomes. However, more research is required to validate results and standardize protocols for routine use. Using powerful machine learning methods, metabolomics can also be used to study neurodevelopment and neuropsychiatric disorders in children. For example, a recent study used an in vitro traumatic brain injury (TBI) model to identify differentially abundant metabolites and affected cellular processes; future research may validate these metabolites and subsequently establish them as biomarkers in a screening panel or as stratifiers for personalized treatments [166]. Metabolomic profiles were also investigated in vivo in pediatric TBI, along with other omics platforms [167,168]. Other studies have investigated metabolomic changes in concussed athletes to develop a robust screening panel to improve the identification of concussions [169,170]. Future advances in metabolomics may lead to affordable bedside tests for detecting biomarkers in children, enhancing diagnosis, management, and prognosis of neurological and neurodevelopmental conditions [5,130,171].

### 7.5. Infectious and Inflammatory Diseases: Sepsis, Infection Biomarkers, COVID-19

Metabolomics enables researchers to identify and understand biomarkers for infectious and inflammatory diseases. This supports earlier diagnosis, better disease classification, and more personalized treatments. In sepsis, especially in newborns, metabolomics can identify metabolic changes that traditional tests may miss. These findings require confirmation in larger, independent studies before they are considered for routine clinical use. Indeed, other studies have employed transcriptomics techniques to identify molecular endotypes in pediatric sepsis that respond differently to treatments such as corticosteroids [172]. Additionally, studies have already investigated metabolomic profiles of pediatric sepsis patients [173,174,175,176,177]. In COVID-19, metabolomics identified differentially abundant metabolites that could classify critically ill COVID-19 patients and healthy controls; specifically, an arginine/kynurenine ratio aided classification of cases versus controls, highlighting the predictive utility of metabolomics profiling. Other studies have also investigated metabolomic profiles in COVID-19, focusing on pediatric populations and pregnant women using single- or multi-omics approaches [178,179,180]. Combining metabolomics with other research methods can further enhance our understanding of diseases and improve the diagnosis and prediction of outcomes for sepsis, infections, and vaccine responses [77,162,181].

### 7.6. Respiratory: Asthma, Cystic Fibrosis

Studies have identified promising metabolomic biomarkers for cystic fibrosis, including increased airway amino acids, small peptides, and alterations in phospholipids and sphingolipids. Shifts in adenosine metabolism, polyamine synthesis, and oxidative stress may also represent potential biomarkers and therapeutic targets, though further validation is required as new modulator therapies are introduced. Non-invasive breath analysis is a promising approach; however, technical challenges persist [182]. In asthma, elevated ceramide glycosyl-n-stearoyl-sphingosine and associations with SNP 17q21, which affects ORMDL3 and sphingolipid synthesis, indicate an early-onset endotype linked to sphingolipid levels. Large pediatric cohort studies have identified and validated five asthma metabo-endotypes based on lipid profiles, providing valuable insights for diagnosis and research [183].

### 7.7. Cardiovascular and Congenital Heart Disease

Studies have applied metabolomics to aid in diagnosing congenital heart disease (CHD), utilizing samples such as maternal serum, urine, and amniotic fluid to identify potential biomarkers. However, the range of known, detectable metabolites remains limited, with inconsistent results, and the underlying mechanisms are not fully understood. Analyses of amniotic fluid from the second and third trimesters using untargeted metabolomic assays were previously performed, and specific metabolic markers of CHD were identified [184]. Untargeted metabolomics remains a valuable tool for uncovering factors involved in the onset of CHD. It offers potential for advancing precision cardiology by distinguishing between individuals with the same genetic mutation who do or do not develop cardiac defects. Identifying affected metabolic pathways may clarify disease mechanisms and reveal new biomarkers. However, CHD-specific metabolomics research is still in its early stages. To fully realize its clinical potential, large multicenter studies are needed.

Arrhythmogenic right ventricular cardiomyopathy (ARVC) is a life-threatening inherited disease that can cause ventricular arrhythmias and sudden cardiac death. Mutations in at least eight genes have been linked to ARVC, including the rare TMEM43 p.S358L variant in males. This mutation is similar to CG8111 p.S333 in *Drosophila melanogaster*, for which metabolomics has shown that S333 is essential for lipid metabolism. Similar lipid-metabolism impairments have been observed in a murine ARVC model characterized by fibrofatty replacement. These findings may help clarify the molecular basis of the TMEM43 p.S358L variant in humans, thus providing another example of the utility of metabolomics in treating human disease [185].

### 7.8. Kidney, Liver, and Other Organ-Specific Disorders

Pediatric renal diseases include a wide range of conditions with lasting effects. Changes in metabolomic profiles have been examined by using advanced analytical tools. Progress in pediatric nephrology metabolomics has enabled the identification of reliable biomarkers, the development of new therapeutic targets, and a better understanding of molecular mechanisms. As kidney disease becomes a more prominent chronic condition, metabolomics offers valuable insights into pediatric renal diseases, including neonatal disorders, kidney injuries, cystic diseases, and congenital anomalies. In these diseases, metabolomics shows promise in assessing physiological status, diagnosing diseases, identifying disrupted pathways, evaluating drug responses, monitoring nutrition, discovering biomarkers, classifying phenotypes, and analyzing sample composition. When large datasets from omics technologies are analyzed with advanced machine learning techniques, they reveal new insights and subtle patterns that are inaccessible to traditional statistical methods. This represents a shift from limited biochemical analysis to comprehensive metabolic profiling, ultimately furthering our understanding of disease and laying the foundation for higher-quality, personalized care [2,5,186].

Many metabolites are identified across different disease conditions. Validated metabolites serve as biomarkers for specific diseases. Table 2 outlines the various disease categories and the metabolites identified within each.

## 8. Multi-Omics Integration of Metabolomics

Although metabolomics offers a comprehensive view of metabolism, multi-omics integration remains essential for personalized medicine. Individual omics technologies, including genomics, transcriptomics, proteomics, and metabolomics, generate comprehensive data; however, they are inherently confined to a single biological layer or domain(e.g., gene expression, protein abundance and modification, small molecules). However, in normal physiological processes, biological layers are highly interconnected and influence one another, this is the central dogma of “systems biology” [201]. As a result, diseases affect multiple biological “omes” or layers by perturbing these interconnected pathways. Finally, integrating multi-omics datasets increases the predictive power of machine learning models for tasks such as subphenotype identification and treatment recommendations [202]. Therefore, the transition from single-omics to integrated multi-omics platforms is a logical, necessary progression, but it also poses significant challenges. The high dimensionality of data (i.e., hundreds to thousands of genes, proteins and metabolites), combined with the variety of data formats across the omics platforms, renders multi-omics integration a non-trivial task. Naturally, integration requires powerful computational approaches; however, when successful, the results are highly informative and relevant. Popular integration methods come in both user-friendly and code-based tools. The aforementioned MetaboAnalyst 6.0 can perform multi-omics integration via its Joint Pathway Analysis module [41]. OmicsAnalyst 2.0 (Xia Lab, McGill University, Ste. Anne de Bellevue, QC, Canada) is another web-based tool for multi-omics integration, providing a simple graphical user interface for combining complex omics datasets [203]. XCMS Online 3.7.1 (Scripps Research, La Jolla, CA, USA), originally a metabolomics data-processing platform, has expanded to include pathway analysis and the integration of genomic and proteomic datasets [204]. Finally, code-based tools such as mixOmics 6.0.0 (Melbourne Integrative Genomics, University of Melbourne, Parkville, VIC, Australia) [205], Multi-Omics Factor Analysis + (MOFA+) (EMBL’s European Bioinformatics Institute (EMBL-EBI), Hinxton, Cambridgeshire, UK) [206], iClusterPlus 1.46.0 (H. Lee Moffitt Cancer Center and Research Institute, Tampa, FL, USA) [207], and SNFtool 2.3.1 (The Hospital for Sick Children (SickKids), Toronto, ON, Canada) [208] are among the most popular frameworks, using languages such as R version 4.5.2 (R Foundation for Statistical Computing, Vienna, Austria) and Python 3.14 (Python Software Foundation, Beaverton, OR, USA). However, the usage of these tools requires higher proficiency in coding and computer skills. Despite the challenges, the integration of multi-omics datasets remains crucial to deepening our understanding of diseases, molecular mechanisms, and characterizing subphenotypes toward the ultimate goal of transitioning to quality, personalized medicine.

## 9. Case Studies and Success Stories

### 9.1. Vignettes Where Metabolomics Changed Diagnosis or Management

One study explored the application of nuclear magnetic resonance (NMR) metabolomic profiling as a promising approach for diagnosing and predicting mortality in pediatric septic shock. It further examined how quantitative metabolomic methods can enhance the clinical evaluation of this life-threatening condition. The methodology employed ^1^H NMR and computational analysis to detect and quantify concentrations of various metabolites in pediatric serum samples. Through targeted profiling, unique spectral intensities of individual metabolites were identified and quantified, enabling comprehensive metabolomic assessment. The findings of this case study identify metabolic changes unique to septic shock and those associated with increased mortality, compared with control pediatric serum samples. The results highlight the potential of metabolomics for early diagnosis and prognosis of septic shock in the pediatric intensive care unit, reinforcing the need for further clinical evaluation [186,209].

A 2023 study investigated metabolic mechanisms in children with medulloblastoma (MB) using advanced mass spectrometry techniques. Researchers identified 25 metabolites with significant differences between MB patients and controls. Functional analysis indicated disruptions in arachidonic acid metabolism, steroid hormone biosynthesis, and folate-related metabolism. The authors concluded that targeting these pathways may reduce MB mortality and that the identified metabolites could serve as effective diagnostic biomarkers [210].

Another study identified plasma biomarkers associated with hepatotoxicity in pediatric patients using an observational case–control design. Metabolomic analysis of 55 children with xenobiotic liver toxicity and 88 healthy controls showed distinct metabolic profiles. Hydroxydecanoylcarnitine, octanoylcarnitine, lysophosphatidylcholine, glycocholic acid, and taurocholic acid emerged as potential biomarkers. Pathway analysis implicated primary bile acid biosynthesis and taurine and hypotaurine metabolism (*p* < 0.05) as significant pathways. Untargeted metabolomic analysis revealed elevated bile acid levels in children with hepatotoxicity. Further research is needed to clarify the role of cytotoxic bile acid accumulation in drug-induced liver injury [211].

Another study highlighted how metabolomic profiling provides real-time insights into graft health, supporting personalized management to improve patient outcomes. The findings demonstrate that non-invasive metabolomics can transform monitoring and treatment for pediatric kidney transplant recipients [212].

### 9.2. Integration of NGS and Metabolomics for Tumor Monitoring

A combined genomic and metabolomic approach, using untargeted metabolomics, identified two new plasma biomarkers for Snyder-Robinson Syndrome and infantile cerebellar-retinal degeneration. Advances in metabolomic databases and processing tools have improved biomarker identification for diagnosing inborn errors of metabolism. Despite these advances, challenges remain, such as limited knowledge of genetic variants, complex data interpretation, and ethical concerns about data use and consent. Overcoming these barriers is crucial to realizing the benefits of genomic newborn screening and promoting equitable access. Technological advancements have reduced clinical report turnaround times, with rapid tests now delivering results in under two weeks. This faster pace is especially valuable in intensive care unit settings, where timely clinical decisions are essential. Multiple studies have shown the cost-effectiveness and clinical utility of rapid whole-exome and whole-genome sequencing in neonatal and pediatric intensive care units. Between 2012 and 2021, 33 clinical studies reported the diagnostic value of first-tier rapid whole-genome sequencing in these environments. With adequate resources, turnaround times can be further reduced; for example, in 2012, disease-causing mutations were identified within 50 h, while a recent 2022 study achieved diagnosis in just 5 h. Continued advancements in genome-based research are improving diagnostics, treatment strategies, and clinical decision-making. The introduction of new processing tools and the expansion of metabolomic databases have enhanced the diagnosis of inherited metabolic disorders, with biomarkers now included in newborn screening protocols. Effective implementation of multi-omics approaches is expected to significantly transform the diagnosis and treatment of patients with undiagnosed rare diseases [213].

### 9.3. Metabolomics and the Microbiome

Another study provides the integration between the gut microbiome, microbial metabolites, and the host metabolome. The findings reveal dysbiosis of gut microbiota, functional impairment, and metabolic disturbances associated with pediatric neurodevelopmental disorders. These results offer a theoretical foundation for the development of microbiota- and metabolite-targeted therapeutic strategies for childhood neurodevelopmental disorders [214].

Untargeted metabolomics has been previously applied to investigate metabolic profiles and identify biomarkers and pathways associated with inherited metabolic diseases. This study sought to identify a distinctive metabolic profile and biomarkers for maple syrup urine disease (MSUD) in newborns using this approach. Dried blood spot samples from 22 MSUD and 22 healthy control newborns were analyzed [215]. The analysis identified 210 altered endogenous metabolites in MSUD newborns, including potential new biomarkers such as L-alloisoleucine, methionine, and lysoPI. The most significantly affected pathways were the ascorbate and aldarate pathways, as well as pentose and glucuronate interconversions, indicating that oxidative and detoxification processes may occur early in life. These results suggest new potential biomarkers and pathways that could support early diagnosis or screening of MSUD in newborns, although further validation is required. The findings provide new insights into the pathogenicity of MSUD and may inform early treatment strategies to enhance health outcomes [215]. Untargeted metabolomic analysis revealed several compounds associated with abnormal flavin adenine nucleotide function, which normalized after riboflavin therapy. This helped confirm the molecular diagnosis and identified candidate biomarkers for targeted monitoring of treatment response. Cases like these demonstrate that untargeted metabolomics can do more than aid diagnosis; it can also help identify biomarkers to guide treatment decisions and monitor treatment response. This approach is relevant not only for patients with inborn errors of metabolism but also for other diseases with significant health and economic impacts, such as cancer, cardiovascular disease, and infectious diseases. Untargeted metabolomics can also support a personalized approach to medicine that considers each patient’s unique situation, including factors like diet, treatment, and environment. Furthermore, advances in single-cell or organelle metabolomics may alter how we treat cancer and enhance our understanding of cellular aging [216].

## 10. Translation to Clinical Practice

### 10.1. Assay Validation and Clinical Lab Standards (CLIA, ISO, GLP)

Assay validation and adherence to clinical laboratory standards ensure that laboratory data are accurate, reliable, and suitable for clinical or regulatory use. International and national guidelines such as CLIA, ISO, and GLP support laboratories in maintaining these standards. The Clinical Laboratory Improvement Amendments (CLIA) of 1988 established a federal regulatory framework for all clinical laboratory testing performed on humans in the United States. CLIA regulations require laboratories to obtain appropriate certification, follow specific quality standards, and undergo regular inspections. The goal is to ensure the accuracy, reliability, and timeliness of patient test results. Different CLIA certificates and regulatory requirements apply based on the complexity and type of testing performed by the laboratory [217].

ISO standards, such as ISO 15189, define requirements for medical laboratories to demonstrate quality and competence. These standards emphasize a robust quality management system, qualified personnel, continuous improvement, and effective international collaboration [218]. Laboratories accredited to ISO 15189 demonstrate their ability to deliver reliable results that support clinical decision-making and global cooperation. Good Laboratory Practice (GLP) is an internationally recognized quality system primarily relevant to non-clinical laboratory studies, including environmental, chemical, and pharmaceutical safety testing. The OECD Principles of GLP define the responsibilities of management, study directors, personnel, and quality assurance, and establish minimum standards for facilities, equipment, documentation, and data integrity. GLP-compliant studies are crucial for regulatory submissions and product safety evaluations; however, they do not apply to studies involving human subjects [219].

Assay validation demonstrates that a laboratory method is effective for its intended purpose by evaluating accuracy, precision, sensitivity, specificity, linearity, and robustness. In clinical diagnostics, this process often includes comprehensive clinical trials to confirm the test’s reliability and utility. Biomarker validation further ensures that both the biomarker and the assay meet specific clinical requirements, which may vary depending on the intended application. By adhering to CLIA, ISO, and GLP standards and rigorously validating their assays, laboratories generate high-quality data that support patient care, fulfill regulatory requirements, and advance scientific research [220].

### 10.2. Regulatory Pathways for Clinical Metabolite Tests/Newborn Screening

Regulatory pathways for clinical metabolite tests and newborn screening are designed to ensure safety, effectiveness, and reliability, particularly for newborns and vary by country. In the United States, the Food and Drug Administration (FDA) oversees these tests, including diagnostic devices, laboratory-developed tests, and in vitro diagnostic (IVD) devices commonly used in metabolite analysis and newborn screening. Manufacturers must demonstrate that clinical metabolite tests are accurate, reliable, and improve health outcomes. They are required to conduct validation studies, adhere to Good Laboratory Practice (GLP), and submit their data to the FDA. The review process may include premarket notification, premarket approval (PMA), or De Novo classification, depending on the test’s novelty or risk level. Newborn screening programs, mandated by state public health authorities and guided by federal recommendations, use highly validated and regulated metabolite tests. These tests must comply with Clinical Laboratory Improvement Amendments (CLIA) and, when applicable, FDA requirements for IVD devices. The regulatory process ensures tests are sensitive and specific enough to detect inborn errors of metabolism and other rare diseases early in life. In summary, the regulatory process for clinical metabolite tests and newborn screening supports innovation while prioritizing patient safety through rigorous evidence of test performance [221].

### 10.3. Workflow Integration and Electronic Health Record (EHR) Incorporation

Integrating multi-omics data with electronic health records is transforming precision medicine. This approach enhances our understanding of diseases and enables more personalized patient care. By combining clinical, molecular, and real-time bio-signal data within EHRs, clinicians and researchers can access comprehensive information and make faster, more informed decisions. A significant advantage of integrating multi-omics and clinical data is the ability to distinguish complex, overlapping diseases into distinct subtypes. This results in more accurate diagnoses, targeted treatments, and deeper insights into disease patterns, particularly in large and diverse patient populations. However, implementing this integration presents significant challenges. Data sources and EHR systems are often incompatible. To ensure smooth data flow, we must standardize formats, adopt consistent terminology, and build robust data pipelines. Clinicians also require user-friendly tools that integrate diverse information and seamlessly fit into their daily workflows. Artificial intelligence and machine learning are playing a key role here. For instance, tools like clinical and omics multimodal analysis enhanced with transfer learning (COMET) (Stanford University School of Medicine, Stanford, CA, USA) utilize large EHR datasets and advanced data fusion methods to facilitate the analysis of smaller omics studies. These approaches enhance predictive power, support new biological discoveries, and enable more detailed patient grouping than traditional methods. Integrating EHRs with multi-omics data and AI has the potential to advance healthcare significantly. Clinicians can access real-time insights into patient risks, disease progression, and treatment effectiveness. However, ongoing attention to data privacy, security, ethics, and staff training remains essential. Collaboration across disciplines will be crucial to the successful implementation of these solutions in routine care [222,223,224,225].

### 10.4. Health Economics: Cost-Effectiveness, Reimbursement

Integrating metabolomics into clinical practice requires clear evidence of cost-effectiveness and a defined reimbursement pathway. Advances in automation and analytical tools are lowering testing costs, making rapid bedside screening for neonatal biofluids increasingly practical. Demonstrating both clinical and economic value is essential for securing reimbursement and encouraging broader adoption. A strong economic case enables innovative, personalized care for neonates and children while supporting healthcare sustainability. Similarly, routine use of multi-omics platforms relies on demonstrated cost-effectiveness. These savings result from economies of scale, greater automation, and improved workflows. Demonstrating these cost reductions, along with improved patient outcomes, strengthens the case for reimbursement and broadens patient access. Ultimately, delivering personalized care at lower costs can transform healthcare and expand access to advanced molecular diagnostics in pediatrics and beyond [5].

### 10.5. Training, Infrastructure, and Capacity Building

Metabolomics is a complex, multidisciplinary field that lacks standardization. Broadly speaking, omics research extends beyond biology into computer science, driven by the characteristics of the data collected and the complexity of the analyses. Therefore, researchers need broad expertise beyond mere biological knowledge, sound judgment, and diverse skills. The code-based nature of omics research remains a limiting factor for researchers lacking computer science expertise; therefore, packaging algorithms and tools into user-friendly programs remains crucial to ensure accessibility of omics research to those without proficiency in computer-related skills such as coding. When code-based approaches are the only option for conducting research, interdisciplinary collaboration between biologists and computer scientists remains paramount for the successful completion of experiments. Overall, effective, practical training and education for researchers are essential to support their development [226].

### 10.6. Barriers to Clinical Translation

A major barrier to clinical implementation is achieving reproducible measurements across laboratories, platforms, and time. Differences in sample handling, extraction protocols, chromatography, mass spectrometric settings, internal standards, and batch correction strategies can lead to site-specific signatures that fail to generalize. Minimum reporting standards and community quality assurance/control (QA/QC) frameworks are increasingly positioned as practical mechanisms to improve transparency, facilitate replication, and enable cross-study comparability [227,228].

Secondly, many metabolomics biomarker candidates stall because translation requires both analytical validation and clinical validation, and clinical validation hinges on demonstrating clinical prediction/utility (i.e., patient-relevant benefit). The metabolome is also highly dynamic and influenced by genetics and environmental exposures (diet, lifestyle, drugs), so generalizability requires validation in independent, heterogeneous (ideally multi-center) cohorts with rigorous control of confounders [229].

Thirdly, clinical deployment requires implementation within an accredited quality system (e.g., CLIA-certified and/or ISO 15189/CAP-accredited laboratories), including documented analytical validation/verification, ongoing quality control, and proficiency testing where applicable. Under CLIA, laboratories performing non-waived testing must establish or verify test performance specifications before reporting patient results and retain documentation to support the accuracy and reliability of those results. Regulatory oversight for laboratory-developed tests has also been unstable: FDA’s 2024 LDT Final Rule proposed a phased approach with targeted enforcement-discretion categories, but the rule was vacated by a federal district court in March 2025, and the FDA later moved to rescind it, creating planning uncertainty for laboratories considering long-term clinical deployment of omics-based assays [230,231].

Finally, even when technical and regulatory barriers are addressed, adoption depends on whether testing improves care and is economically sustainable. Health-economic evaluation for diagnostics is methodologically distinct from therapeutics and often requires modeling beyond analytical accuracy to estimate downstream clinical outcomes and resource use. Payers typically expect evidence of clinical utility and economic value (e.g., avoided admissions, reduced diagnostic odysseys, improved seizure control through targeted interventions), and implementation must fit clinical workflows (turnaround time, confirmatory testing, clinician interpretation support, and reporting standards) [232].

## 11. Discussion

MS-based metabolomics is crucial in pediatric research; however, challenges with reproducibility and standardization persist. Variability in sample handling, processing, and calibration complicates cross-study comparisons [92,97,98,99]. Consistent protocols such as ISO 23118 and CLSI EP28-A3c enhance data quality and reliability [91,233]. Standardizing calibration, reference materials, and quality control samples support clinical implementation and improves data accessibility and reuse. Integrating clinical data with omics databases, supported by EHR systems and regulations like CLIA and ISO 15189, ensures traceability and validation for clinical use [92,138,220,222,234]. Pooled quality control samples and interlaboratory ring trials enable laboratories to benchmark their performance and advance biomarker discovery [98,103]. Persistent differences in analytical platforms, sample types, and computational methods present ongoing challenges. Addressing these issues will require a global consensus on pediatric-specific protocols, age-adjusted normalization, and free availability of reference datasets. Ethical, legal, and social considerations, including informed parental consent, privacy, and equitable access, must also be taken into account. Emerging technologies, such as high-resolution mass spectrometry, machine learning, and single-cell metabolomics, are making large-scale analysis and clinical applications more feasible. Expanding metabolomics globally will depend on improved infrastructure, enhanced training, and international collaboration. Despite progress, variability, challenges in data interpretation, and the lack of standard pediatric datasets continue to limit clinical adoption. Ongoing innovation, collaboration, and robust ethical oversight are essential for advancing pediatric metabolomics.

## 12. Conclusions

MS-based metabolomics is advancing pediatric health by enabling the early detection of metabolic disorders, tracking developmental progress, and identifying disease biomarkers. Combined with genomics, transcriptomics, and proteomics, it supports more precise and preventive care. However, challenges remain, including the need to create age- and sex-specific reference values, standardize methods, and ensure the ethical use of data. With stringent quality control standards such as ISO, CLIA, and GLP, and the incorporation of AI-powered multi-omics analyses into electronic health records, alongside emerging methods such as single-cell metabolomics, this field is set to become crucial for pediatric diagnostics and personalized medicine worldwide.

## Figures and Tables

**Table 2:** Representative metabolomic biomarkers across pediatric disease categories and typical sample matrices.

Disease Categories	Key Metabolites Identified	Associated Diseases	Sample Origin (Matrix)	References
**Newborn screening and IEM**	Amino acids (Phe, Tyr, Met, Arg, Leu), Citrin, C0, C2, C3, C4, C5, C8, C5-OH	PKU, Hypermethioninemia, MSUD, CIT-I, Tyrosinemia, PCD, MADD, MCAD, HCY, MMA	DBS/whole blood	[187]
**Infectious and inflammatory diseases**	Elevated glucose, lactate, phosphoenolpyruvic acid, ketobutyric acid, propionic acid, TCA cycle metabolites	HIV	Plasma/serum	[188,189,190,191,192]
	ApoB100/Apo-A1 ratio, triglycerides, and GlycA decreased.	SARS-CoV-2	Plasma/serum	
	N-acetylneuraminate, quinolinate, pyridoxate, D-mannose and D-arabinose, α-mycolic acid, methoxy-mycolic acids	Tuberculosis	Plasma/serum	
	Decreased indole propionate, glutamate, arginine, and glutamine. Increased levels of kynurenate.	Malaria	Plasma/serum	
**Metabolic and Endocrine Disorders**	Increased BCAAs (leucine, isoleucine, and valine), glutamic acid, glutamine, and ketoleucine. Low BMI, and high AAAs, Met, Phe, Lys, low Gly levels	Type 2 diabetes	Plasma/serum	[193,194]
	Increased urinary methyl histidine concentration. 1-methylhistidinuria, increased 1-MHis excretion.	NAFLD	Serum/plasma (±liver tissue)	
	Decreased levels of proline, methionine, and glutamine. Increased medium and long-chain AcylCN, neutral BCAA levels	Obesity	Plasma/serum (±urine)	
**Neurological and Neurodevelopmental Disorders**	Xylitol, Phosphoric acid, 1-methylhydantoin, Pyruvic acid, 2-Ketoglutaric acid, Allothreonine,	Cerebral Palsy	Plasma/serum (±CSF)	[195,196,197,198]
	FAHFA (18:1(9Z)/9-O-18:0), DL-2-hydroxystearic acid, and 7(S),17(S)-dihydroxy-8(E),10(Z),13(Z),15(E),19(Z)-docosapentaenoic acid	ASD	Urine and plasma/serum	
	P-cresol, p-cresyl sulphate, indole, indoxyl-sulphate, decreased tryptophan and serotonin, increased KYNA, glyceryl 1-1-palmitate, glyceryl 1-1-stearate level, decreased malic acid, and aconitic acid.	Epilepsy	Plasma/serum (±CSF)	
**Cancers**	1-Methylhistidine, 2-Ketobutyric acid, Deoxyuridine, 4-Pyridoxic acid, α-Ketoisovaleric acid, γ-Glutamylglutamine, Allantoin	Leukemia	Plasma/serum (±bone marrow)	[199]
**Cardiometabolic Health**	Ala, aspartic acid, creatinine, glutamic acid, glycine, Leucine, and Phe	cardiac dysfunction	Plasma/serum	[200]
**Respiratory and allergy**	High levels of 4-hydroxybutyrate, lactate, cis-aconitate, 5-HIAA, taurine, trans-4-hydroxy-l-proline, alanine, glycerol, arginine, sphingolipid, γ-glutamylcysteine	Childhood asthma	Exhaled breath condensate and plasma/serum	[60,183]
	Butyric acid, β-Alanine, Methylamine, Inosine, Acetic acid, Isovaleric acid, Phenylalanine, Acetylcarnitine,	Allergic respiratory rhinitis	Nasal secretions/lavage and plasma/serum	
**Kidney and Renal Disorders**	Increased serum levels by 50%, NGAL, osteopontin (OPN), and cystatin C (CysC)	AKI	Urine and plasma/serum	[186]
	Increased urinary level of N-acetylasparagine, betaine, uric acid, and hypoxanthine, and decreased levels of TMAO	Renal Dysplasia	Urine (±plasma/serum)	
	Increased level of glycine, citrulline, ADMA, and SDMA) while dimethylglycine	CKD	Urine and plasma/serum	
	Decreased bilirubin level	Kidney Stones	Urine	

**Abbreviations:** 1-MHis (1-methylhistidine), 5-HIAA (5-hydroxyindoleacetic acid), AAA (aromatic amino acids), AcylCN (acylcarnitines), AKI (acute kidney injury), Ala (alanine), ApoA1 (apolipoprotein A1), ApoB100 (apolipoprotein B100), Arg (arginine), ASD (autism spectrum disorder), BCAA (branched-chain amino acids), BMI (body mass index), C0 (free carnitine; C0 acylcarnitine), C2 (acetylcarnitine; C2 acylcarnitine), C3 (propionylcarnitine; C3 acylcarnitine), C4 (butyrylcarnitine; C4 acylcarnitine), C5 (isovalerylcarnitine; C5 acylcarnitine), C5-OH (3-hydroxyisovalerylcarnitine; C5-hydroxy acylcarnitine), C8 (octanoylcarnitine; C8 acylcarnitine), CIT-I (citrullinemia type I), CSF (cerebrospinal fluid), CysC (cystatin C), DBS (dried blood spot), FAHFA (fatty acid esters of hydroxy fatty acids), Gly (glycine), GlycA (glycoprotein acetylation), HCY (homocystinuria), HIV (human immunodeficiency virus), IEM (inborn errors of metabolism), KYNA (kynurenic acid), Leu (leucine), Lys (lysine), MADD (multiple acyl-CoA dehydrogenase deficiency; glutaric acidemia type II), MCAD (medium-chain acyl-CoA dehydrogenase deficiency), Met (methionine), MMA (methylmalonic acidemia), MSUD (maple syrup urine disease), NAFLD (non-alcoholic fatty liver disease), NGAL (neutrophil gelatinase-associated lipocalin), OPN (osteopontin), PAHD (phenylalanine hydroxylase deficiency; phenylketonuria), PCD (propionyl-CoA carboxylase deficiency; propionic acidemia), Phe (phenylalanine), PKU (phenylketonuria), SARS-CoV-2 (Severe Acute Respiratory Syndrome Coronavirus 2), TCA (tricarboxylic acid cycle), Tyr (tyrosine).

## Data Availability

No new data were created or analyzed in this study. Data sharing is not applicable to this article.
